# Neural Decoding of Multi-Modal Imagery Behavior Focusing on Temporal Complexity

**DOI:** 10.3389/fpsyt.2020.00746

**Published:** 2020-07-30

**Authors:** Naoki Furutani, Yuta Nariya, Tetsuya Takahashi, Haruka Ito, Yuko Yoshimura, Hirotoshi Hiraishi, Chiaki Hasegawa, Takashi Ikeda, Mitsuru Kikuchi

**Affiliations:** ^1^ Department of Psychiatry and Neurobiology, Graduate School of Medical Science, Kanazawa University, Kanazawa, Japan; ^2^ Faculty of Medicine, The University of Tokyo, Tokyo, Japan; ^3^ Research Center for Child Mental Development, Kanazawa University, Kanazawa, Japan; ^4^ General course, Sundai-Kofu High School, Kofu, Japan; ^5^ Institute of Human and Social Sciences, Kanazawa University, Kanazawa, Japan; ^6^ Department of Biofunctional Imaging, Preeminent Medical Photonics Education & Research Center, Hamamatsu University School of Medicine, Hamamatsu, Japan

**Keywords:** expanded multiscale entropy (expMSE), magnetoencephalography (MEG), mental imagery, neural decoding, multivariate pattern analysis (MVPA), modality specific-regions, supramodal regions, convolutional neural network (CNN)

## Abstract

Mental imagery behaviors of various modalities include visual, auditory, and motor behaviors. Their alterations are pathologically involved in various psychiatric disorders. Results of earlier studies suggest that imagery behaviors are correlated with the modulated activities of the respective modality-specific regions and the additional activities of supramodal imagery-related regions. Additionally, despite the availability of complexity analysis in the neuroimaging field, it has not been used for neural decoding approaches. Therefore, we sought to characterize neural oscillation related to multimodal imagery through complexity-based neural decoding. For this study, we modified existing complexity measures to characterize the time evolution of temporal complexity. We took magnetoencephalography (MEG) data of eight healthy subjects as they performed multimodal imagery and non-imagery tasks. The MEG data were decomposed into amplitude and phase of sub-band frequencies by Hilbert–Huang transform. Subsequently, we calculated the complexity values of each reconstructed time series, along with raw data and band power for comparison, and applied these results as inputs to decode visual perception (VP), visual imagery (VI), motor execution (ME), and motor imagery (MI) functions. Consequently, intra-subject decoding with the complexity yielded a characteristic sensitivity map for each task with high decoding accuracy. The map is inverted in the occipital regions between VP and VI and in the central regions between ME and MI. Additionally, replacement of the labels into two classes as imagery and non-imagery also yielded better classification performance and characteristic sensitivity with the complexity. It is particularly interesting that some subjects showed characteristic sensitivities not only in modality-specific regions, but also in supramodal regions. These analyses indicate that two-class and four-class classifications each provided better performance when using complexity than when using raw data or band power as input. When inter-subject decoding was used with the same model, characteristic sensitivity maps were also obtained, although their decoding performance was lower. Results of this study underscore the availability of complexity measures in neural decoding approaches and suggest the possibility of a modality-independent imagery-related mechanism. The use of time evolution of temporal complexity in neural decoding might extend our knowledge of the neural bases of hierarchical functions in the human brain.

## Introduction

In recent years, neural decoding research has progressed along with the expansion of machine learning (ML). Neural decoding has also been applied for interpreting mental states (1, 2) and for treating various psychiatric disorders with neurofeedback (3–12). With the development of deep learning, decoding performance improvement is accelerating. One successful method is that of convolutional neural networks (CNNs), which are categorized as multivariate pattern analysis (MVPA) and which learn complex features using small filters to learn local patterns and process them through multiple layers. Initially, CNN progressed in the image recognition field, but it has been applied recently in the neuroimaging field (13, 14). Additionally, growing interest exists in interpreting trained models (15). This avenue of research is extremely important for neuroimaging because it helps to elucidate features that the model uses to distinguish the classes. However, the neuroimaging devices have their own characteristics. Among neuroimaging devices, electroencephalography (EEG) and magnetoencephalography (MEG) directly measure brain activity with excellent temporal resolution, thereby yielding insight into temporal dynamics within physiologically relevant frequency ranges.

However, despite remarkable progress in ML, some room exists for improving preprocessing before ML. At present, raw data and band power are used mainly as inputs in E/MEG-based neural decoding. Comparably to raw data, band power improves decoding performance by decomposing the frequency information in advance (14, 16). However, some other analytical methods are useful for evaluating both normal and pathological brain states. For example, temporal complexity of single time series have being studied. Neural oscillations are assumed to be affected by past neuronal processes on various time scales through feedback loops at multiple hierarchical levels of cortical processing (17). This history effect has been well studied as temporal dynamics using multiscale entropy (MSE), which calculates the sample entropy (SampEn) on multiple time scales. Actually, MSE has been applied with great benefit to various neuroimaging devices such as E/MEG and functional magnetic resonance imaging (fMRI) (18, 19). Moreover, it has contributed to elucidation of the neural bases of many psychiatric disorders and conditions, including schizophrenia (20), Alzheimer’s disease (AD) (21–24), autism spectrum disorder (ASD) (25–27), attention-deficit hyperactivity disorder (28), and aging (29, 30). One difficulty, however, is that conventional MSE approaches describe comprehensive unpredictability in a time series irrespective of their diverse information. Because the frequency, amplitude, and phase of E/MEG data are thought to differ in terms of their underlying neural functions (31–34), analyzing decomposed neural signals into frequency, amplitude, and phase might add other directions for elucidating details of neural functions. For example, Ghanbari et al. (27) reported that frequency-decomposed MSE extracts some characteristic features of ASD. Our earlier study similarly revealed alterations of the amplitude and phase MSE in AD patients (Furutani et al., submitted). Another issue is that conventional complexity measures such as SampEn and approximate entropy (ApEn) compute a single value from a time series (35). Although these are extremely useful for representing the time series complexity, clinical data tend to have a small sample size, making them difficult to use for ML as they are. Therefore, we propose a new complexity measure: expanded SampEn (expSampEn). Complexity analyses fundamentally use information theory to evaluate the bias of the probability distribution. Subsequently, the expSampEn evaluates the bias of each time point. In other words, we simply skipped the averaging in the SampEn and ApEn algorithm and obtained a complexity value in the form of a time series (see *expMSE*). Consequently, we used the expanded MSE (expMSE) of decomposed signals as inputs for neural decoding.

Mental imagery is a behavior with various modalities, such as visual, auditory, and motor, that has a multifaceted association with psychiatric disorders. For example, the mental imagery capabilities are altered in autism spectrum disorder (ASD) patients (36), concern-related images are repeated in patients with post-traumatic stress disorder (PTSD) and social phobia (37); negative mental imagery causes distress and strongly affects various psychiatric disorders (38). Mental imagery has also been used in psychotherapy and neurofeedback to treat psychiatric disorders (3, 38). Therefore, it is expected to be important to investigate the neural basis of mental imagery for the treatment of psychiatric disorders and for understanding their pathophysiology. The neural mechanisms of mental imagery have been discussed in terms of modality-specific regions and supramodal imagery-related regions (39). Although primary sensorimotor cortices are often active during mental imagery, their activities might not be fundamentally important. Reportedly, activities in the primary sensorimotor cortices during mental imagery are lower than during perception or execution. They depend on the task intensity of visual imagery (VI) (38, 40) and motor imagery (MI) (41–44). However, other modality-specific regions adjacent to primary sensorimotor cortices, including auditory associative areas (39) and premotor and supplementary motor areas (39, 42, 45), are activated during both imagery and non-imagery (i.e. execution or perception) of each modality. Visual associative areas are divided further into several subtypes depending on the type of VI (39). In addition, although these associative regions are active during both imagery and non-imagery, the connectivity pattern among these regions is altered (46). Furthermore, supramodal imagery-related regions have been reported, including the prefrontal (PFC) and parietal cortex (3, 38, 39, 41, 42, 45, 47, 48), which are regarded as sending top-down inputs to the modality-specific areas (38, 46–48). However, only three reports of the relevant literature describe studies that have examined the multimodal imagery-related brain activities directly, all are of fMRI studies of VI and auditory imagery (AI) (49–51).

In summary, this study’s aims are two-fold: 1) achieve CNN decoding with the expMSE; and 2) achieve hierarchical task decoding to examine supramodal and modality-specific neural oscillations. For this study, we measured MEG in healthy participants performing hierarchical multimodal tasks (visual/motor × imagery/non-imagery tasks), calculated the expMSE, and applied it to CNN decoding.

## Methods

### Participants

This study examined eight healthy participants [S0–S7; 4 male, age 27.1 ± 6.2 years (mean ± SD), 1 left-handed]. All participants were native Japanese speakers reporting no prior or existing psychiatric, neurological, or medical illness. Participants were screened with a structured clinical interview for Diagnostic and Statistical Manual of Mental Disorders (DSM)-IV-TR (52) to confirm a lack of history of personal psychiatric illness. All participants agreed to participate in the study with full knowledge of the experimental characteristics of the research. After a complete explanation of the study, written informed consent was obtained before the start of the experiment. The ethics committee of Kanazawa University Hospital approved the study methods and procedures.

### Tasks and Procedures

All participants underwent MEG examination while performing several tasks. To obtain a diverse distribution of brain activity data, we defined 12 multimodal tasks: visual perception (VP, observing a grayscale picture of Ichiro Suzuki, a famous baseball player); visual imagery (VI, imagining the presented picture); auditory perception (AP, listening to a simple melodic line from ‘Dance of the Four Swans’); auditory imagery (AI, imagining the presented music); motor execution (ME, moving the right index finger); motor imagery (MI, imagining the finger motion); visual imagery 2 (VI2, imagining someone else exercising); motor imagery 2 (MI2, imagining oneself exercising); auditory perception 2 (AP2, listening to sounds in consonance), auditory perception 3 (DL, listening to sounds in dissonance); visual perception 2 (VP2, observing a color picture of a happy individual); and visual perception 3 (VP3, observing a color picture of a sad individual). They completed eight trials × 3 sessions. To acquire a stable index, the tasks were performed successively in a fixed order as described above. Each trial included 6 s of ‘rest’ and 6 s of the ‘task’. Each task was started and stopped in conjunction with an acoustic stimulus. In each of the three sessions, a total of eight trials × 12 tasks were performed. The first session was performed as a practice session. The second and third sessions were analyzed. Sufficient breaks were given between the sessions to prevent fatigue. During each task, the participants opened their eyes and looked at a display. In addition, the first of the eight trials for each task was excluded from analyses because there was no pre-task break. The number of trials for the analyses was 14 (7 trials × 2 sessions) per task.

### MEG Recording

Magnetic fields were measured using a whole-head system for adults at the Laboratory of Yokogawa Electric Corp. in Japan. This system (MEGvision PQA160C; Yokogawa Electric Corp., Japan) consisted of 160 channels. Magnetic fields were sampled at 2,000 Hz per channel (bandpass 0.16–500 Hz). The T1-weighted magnetic resonance imaging (MRI) images were acquired (Sigma Excite HD 1.5 T; GE Yokogawa). All participants had pointed spherical lipid markers placed at the five MEG fiduciary points to enable superposition of the MEG coordinate system on the MRI. The MRI consisted of 166 sequential slices of 1.2 mm, with resolution of 512 × 512 points in a field of view of 261 × 261 mm. Individual cortex envelopes were extracted using FreeSurfer 5.1 for cortical surface-based analysis (number of voxels: 15,000) (53, 54).

### Data Preprocessing

**Figure 1:**
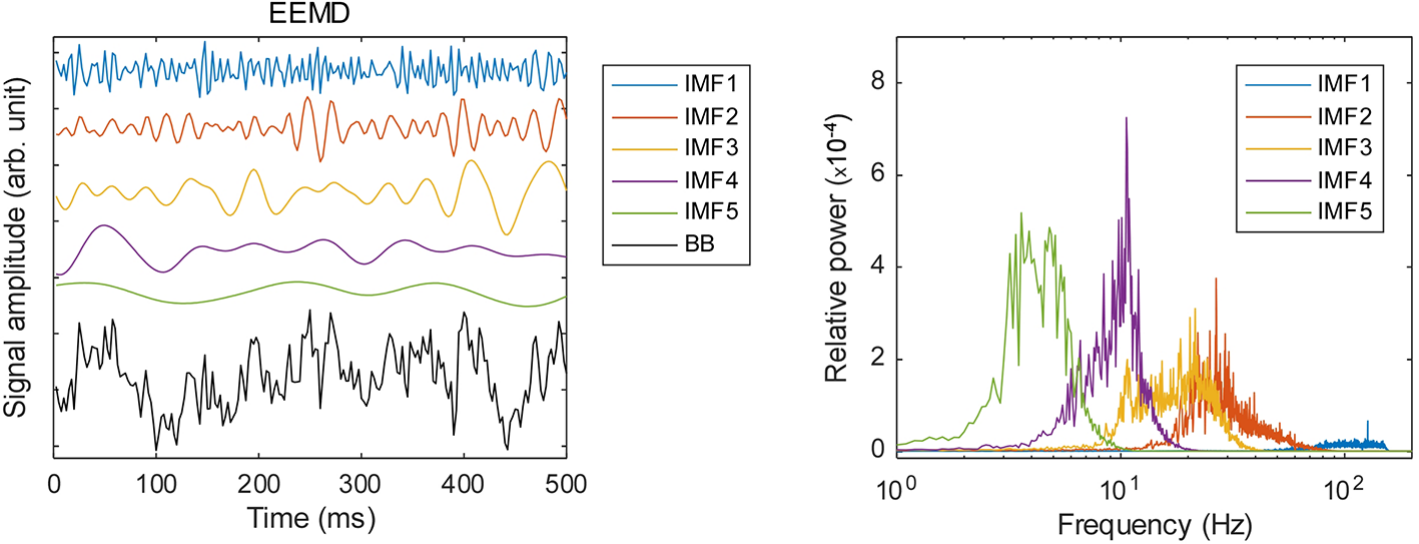
Frequency decomposition by ensemble empirical mode decomposition (EEMD): left panel, examples of the decomposed time series; right panel, relative power spectral densities of the respective IMFs.

Preprocessing of the MEG data presented in this section was conducted using software [MATLAB; The Mathworks Inc., Natick, MA and Brainstorm (55)]. The MEG data were resampled at 400 Hz with 150 Hz low-pass and 60 and 120 Hz notch filters. Subsequently, the data were cleaned using a signal-space projection (SSP) algorithm for the removal of blink and heartbeat signals. After the magnetic field data were transformed into a source time series using a weighted minimum norm estimation (wMNE) algorithm (56–58), they were averaged within each of the 68 regions of the Desikan–Killiany brain atlas (59). The source time series were decomposed into amplitude and phase of sub-band frequencies using ensemble empirical mode decomposition (EEMD) and Hilbert spectral analysis (HSA) as reported by Huang et al. (60, 61). We implemented EEMD with addition of white noise at 0.2 standard deviations of amplitude relative to the original source time series. Then we calculated an average of 200 ensembles as the IMF. **Figure 1** portrays the relative power spectral densities of the decomposed time series. Actually, EEMD is an adaptive method that differs from many other frequency decomposition methods. Although that feature represents an advantage of the EEMD, the IMF frequency varies according to the sampling rate (SR) and low pass filtering (LPF) of the time series. Given the conditions used for this study (sampling frequencies 400 Hz; LPF, 150 Hz), the peak frequencies of IMF 1–5 are approximately >100 Hz, 40 Hz, 20 Hz, 10 Hz, and 4 Hz. Therefore, we analyzed IMF 2–4 for the remainder of the analyses and respectively designated them as gamma, beta, and alpha bands. These IMFs were processed further using Hilbert spectral analysis (HSA) and were decomposed into an amplitude and phase time series.

### Inputs

#### expMSE

To assess the temporal changes of the complexity, we modified existing complexity measures. ApEn and SampEn are well known to represent the temporal complexity of the time series. One report of the literature by Porta et al. (35) proposed local sample entropy (LSampEn) as a modified version of them. Each of ApEn, SampEn, and LSampEn represents unpredictability of the value of the (*m+*1)-th time point from 1 to *m* time points. As described by Porta et al. (35), ApEn, SampEn, and LSampEn differ in the stage of averaging over time. Consider a time series ={*x*_*n*_, *n*=1,…,*N* } when *n* represents each time point and *N* denotes the total length. Define *x_n_* as the current value of *x*, and $x_{n}^{-}=\left[x_{n-m}\cdots x_{n-1}\right]$ is the *m*-dimensional past values; ***x***_*n*_=[*x*_*n*−*m*_⋯*x*_*n*_ ] is the (*m+*1)-dimensional vector obtained by concatenating *x_n_* to $x_{n}^{-}$. Define $p\left(x_{n}|x_{n}^{-}\right)$ as the conditional probability that the current value is *x_n_* given past values $x_{n}^{-}$, and define *p*(***x***_*n*_) and $p\left(x_{n}^{-}\right)$ respectively as the joint probabilities that (*m+*1)-dimensional and *m*-dimensional vectors described earlier are ***x***_*n*_ and $x_{n}^{-}$. Then, ApEn, SampEn, and LSampEn are represented as shown below.

$$\text{ApEn}=-\langle \log p\left(x_{n}|x_{n}^{-}\right)\rangle =-\langle \log\frac{p\left(x_{n}\right)}{p\left(x_{n}^{-}\right)}\rangle$$

$$\text{SampEn}=-\log\frac{\langle p\left(x_{n}\right)\rangle }{\langle p\left(x_{n}^{-}\right)\rangle }$$

$$\text{LSampEn}=-\log\langle p\left(x_{n}|x_{n}^{-}\right)\rangle$$

Therein, <∙> represents the average over time. They can be summarized briefly as follows: ApEn represents the entropy computed at each time point and then averaged; SampEn represents the average of each probability before computing entropy; and LSampEn is a value representing the entropy after averaging the probability distribution. For the present study, expSampEn is defined as the time evolution of complexity without performing averaging over time.

$$\text{expSampEn }\left(n\right)\;=-\log p\left(x_{n}|x_{n}^{-}\right)$$

This idea was inspired by the modified version of mutual information: local mutual information (62). For this study, four tasks were analyzed (VP, VI, ME, and MI), but all tasks were used as the index for calculating the expSampEn. We observed the expSampEn on various time scales (i.e. expMSE) in the same way as MSE. We used *m* = 2 and *r* = 0.2 to calculate the entropy (23). Considering the frequency of each IMF, five time scale factors (TSFs) were used (gamma—2, 4, 8, 16, and 32; beta—4, 8, 16, 32, and 64; alpha—8, 16, 32, 64, and 128). After the expMSE analysis, the SR was adjusted to 0.64 s for ML.

One important shortcoming of ApEn is that the log contents often become 0 because the entropy is calculated at each time point (35). We also compute the entropy at each time point. Therefore, the expSampEn cannot be calculated at some time points. We have taken two solutions to this problem. First, we increased the index size for calculating the complexity. The MEG data were measured for 20 min at 400 Hz. There were approximately 500,000 time points in each IMF time series at each region. As described above, the maximum TSF is 128, so the minimum index size for the complexity measures is approximately 4,000 points. This is insufficient for the complexity measure. Therefore, we concatenated the indices of all 68 brain regions; then, we coarse-grained it to obtain the index size of 250,000–500,000 points for each TSF because the large index size can be computationally intensive. Consequently, the expSampEn was computed using the standardized index across regions. Next, we linearly interpolated the time points that remain uncomputable. The relations between SampEn, ApEn, and expSampEn are presented in **Tables 1**–**4**.

**Table 1:** Relation between the expMSE and SampEn of the amplitude in all 68 regions in subject S0 (Pearson’s correlation, mean ± SD).

	TSF A	TSF B	TSF C	TSF D	TSF E
Gamma	0.86 ± 0.10	0.83 ± 0.11	0.79 ± 0.11	0.75 ± 0.10	0.65 ± 0.12
Beta	0. 83 ± 0.10	0.82 ± 0.10	0.76 ± 0.12	0.66 ± 0.15	0.52 ± 0.20
Alpha	0.84 ± 0.07	0.77 ± 0.09	0.68 ± 0.14	0.50 ± 0.17	0.30 ± 0.22

The expMSE were averaged over 51.2 s. SampEn were calculated for the same time window. TSFs A–E are 2, 4, 8, 16, and 32 for gamma, 4, 8, 16, 32, and 64 for beta, 8, 16, 32, 64, and 128 for alpha.

TSF, time scale factor.

**Table 2:** Relation between the expMSE and ApEn of the amplitude in all 68 regions in subject S0 (Pearson’s correlation, mean ± SD).

	TSF A	TSF B	TSF C	TSF D	TSF E
Gamma	0.91 ± 0.06	0.88 ± 0.07	0.78 ± 0.11	0.46 ± 0.24	−0.12 ± 0.29
Beta	0.92 ± 0.04	0.82 ± 0.10	0.34 ± 0.33	−0.28 ± 0.34	−0.57 ± 0.20
Alpha	0.90 ± 0.05	0.65 ± 0.14	−0.18 ± 0.28	−0.66 ± 0.11	−0.63 ± 0.15

The expMSE were averaged over 51.2 s. ApEn were calculated for the same time window. TSFs A–E are 2, 4, 8, 16, and 32 for gamma, 4, 8, 16, 32, and 64 for beta, 8, 16, 32, 64, and 128 for alpha.

TSF, time scale factor.

**Table 3:** Relation between the expMSE and SampEn of the phase in all 68 regions in subject S0 (Pearson’s correlation, mean ± SD).

	TSF A	TSF B	TSF C	TSF D	TSF E
Gamma	0.57 ± 0.29	0.32 ± 0.23	−0.19 ± 0.25	0.04 ± 0.18	0.07 ± 0.23
Beta	0.56 ± 0.21	0.43 ± 0.17	0.17 ± 0.29	0.08 ± 0.19	0.02 ± 0.18
Alpha	0.83 ± 0.11	0.59 ± 0.24	0.30 ± 0.27	0.07 ± 0.21	0.00 ± 0.19

The expMSE were averaged over 51.2 s. SampEn were calculated for the same time window. TSFs A–E are 2, 4, 8, 16, and 32 for gamma, 4, 8, 16, 32, and 64 for beta, 8, 16, 32, 64, and 128 for alpha.

TSF, time scale factor.

**Table 4:** Relation between the expMSE and ApEn of the phase in all 68 regions in subject S0 (Pearson’s correlation, mean ± SD).

	TSF A	TSF B	TSF C	TSF D	TSF E
Gamma	0.79 ± 0.17	0.47 ± 0.22	−0.14 ± 0.23	0.06 ± 0.19	0.04 ± 0.21
Beta	0.73 ± 0.18	0.51 ± 0.19	0.18 ± 0.24	0.05 ± 0.18	−0.01 ± 0.18
Alpha	0.88 ± 0.09	0.67 ± 0.21	0.30 ± 0.27	0.05 ± 0.21	−0.04 ± 0.17

The expMSE were averaged over 51.2 s. ApEn were calculated for the same time window. TSFs A–E are 2, 4, 8, 16, and 32 for gamma, 4, 8, 16, 32, and 64 for beta, 8, 16, 32, 64, and 128 for alpha.

TSF, time scale factor.

We performed the above calculations for each amplitude and cosine of phase time series and obtained 68 regions × 3 frequency bands × 2 components × 5 TSFs decomposed expMSE time series. We reshaped it into 68 × 30 time series.

#### Raw Data and Band Power

To compare the decoding performance, we also used raw data and band power as inputs. As shown in **Figure 1**, IMF2 (gamma) is approximately less than 80 Hz. Therefore, we downsampled the raw data to 200 Hz. We also computed the band power time series and then coarse-grained it to SR of 0.64 s to align with the expMSE. Therefore, we obtained 68 frequency bands × 3 regions time series.

#### Data Cropping

Additionally, we adopted a cropping strategy (13, 63, 64). The task period is 6 s. Therefore, we obtained nine time points at SR of 0.64 s. We chose four of these nine points and averaged them across all combinations, yielding 126 crops (= _9_ *C*_4_) per trial. Consequently, 12 tasks × 14 trials × 126 crops were obtained both for the expMSE and the band power time series. However, 134 crops were obtained for the raw data with a sliding 2 s window (400 time points).

### Decoding

CNN decoding in this section was conducted using Keras 2.3.1 (65).

#### CNN Model

For this study, we adopted a CNN model: a deep neural network that can learn local patterns in data using convolutional filters. Although CNN has made remarkable progress, especially in computer vision tasks, it has also been successful in recent years for neural decoding (13, 14, 66).

**Figure 2:**
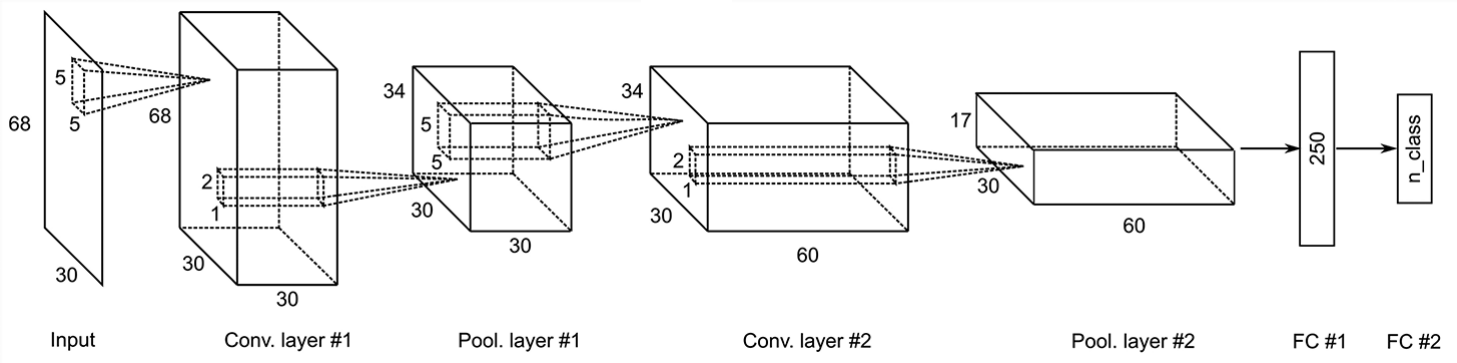
Convolutional neural network (CNN) architecture for the expMSE, including Input, Conv, Pool, and FC layers. The equivalent model was also used for the band power, but the input was reduced to 68 × 3. Therefore, only the convolutional filter was reduced to 5 × 2. Conv., convolutional; Pool, = max pooling; FC, fully connected.

As described earlier, we used some analytic methods for feature extraction. As reported by Tayeb et al. (14), when using *a priori* feature extraction, shallow CNN showed high decoding accuracy comparable to that of deep CNN. Because we applied EEMD, HSA and complexity analysis to obtain the inputs, we adopted a shallow CNN (**Figure 2**). The inputs of expMSE were fed into our CNN model, which includes two convolution blocks, followed by a dense softmax classification layer. Each convolution block includes one convolutional layer, batch normalization, and a max-pooling layer. An exponential linear unit (ELU) is used as the activation function. As the optimization method, we adopted Adam (67), a stochastic optimization method, together with an early stopping method. Preliminary experiments showed better decoding performance when using small pooling filters (data not shown), probably because neural decoding has less need for shift-invariance, which is one important benefit of pooling, than image recognition. Furthermore, Schirrmeister et al. (13) used a large filter as a spatial filter in the first layer. However, a smaller filter, like that used in the present study, tended to be more accurate (**Supplementary Table 1**). Therefore, we used 5 × 5 convolutional filters. For comparison, decoding was also performed using raw data and band power as inputs. Also, equivalent models were used. The input sizes were 68 × 400 and 68× 3. The convolutional filters were 5 × 10 and 5 × 2, respectively, for raw data and band power.

#### Cropped Training

As explained in *Data Cropping*, 126 crops were obtained per trial (134 crops for raw data). This led to multiple label predictions per trial. The average of these predictions was used as the final prediction for the trial during the test phase. During training, we computed a loss for each prediction.

#### Intra-Subject and Inter-Subject Decoding

We performed decoding with intra-subject and inter-subject designs. For intra-subject decoding, 14 trials were divided into four groups (three or four trials per group). The decoding performance was examined in 12 combinations of train:validation:test = 2:1:1. For inter-subject decoding, all 14 trials of one subject were used as test data. Seven trials of the remaining subjects were assigned to training and validation data.

#### Statistics

Differences in decoding performance were examined using paired *t*-tests (two-tailed).

#### Visualization

In addition to identification of the features used for the classification, we calculated a gradient-based sensitivity map of the model for each feature map (68, 69). This method has been studied intensively in the field of image recognition. It is applicable to neural networks. We applied it to neural decoding. Letting *S_c_*(*x*) be the score of the class *c* computed by the classification layer of the CNN for an input *x*, then the final classification *class*(*x*) can be represented as

$$class\left(x\right)=\;{\text{argmax}}_{c}\;S_{c}\left(x\right)$$

**Figure 3:**
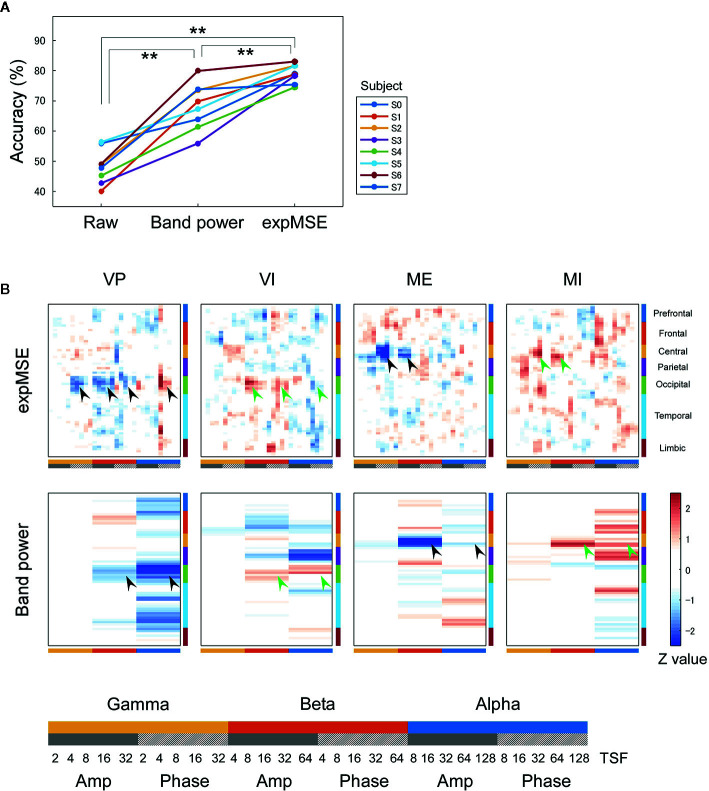
Intra-subject decoding of each task. **(A)** Decoding accuracies compared among raw data, band power and expMSE. Each color corresponds to a subject; **p < 0.01. **(B)** Task-related sensitivity maps of the example (subject S4). Upper and lower panels respectively portray maps for the expMSE and band power (VP, visual perception; VI, visual imagery; ME, motor execution; MI, motor imagery; black arrow, modality-specific sensitivity; green arrow, inverted sensitivity in the modality-specific regions). At the bottom are details of the horizontal axes: frequency × component (Amp/Phase) × TSF; Amp, amplitude expMSE; Phase, phase expMSE.

**Figure 4:**
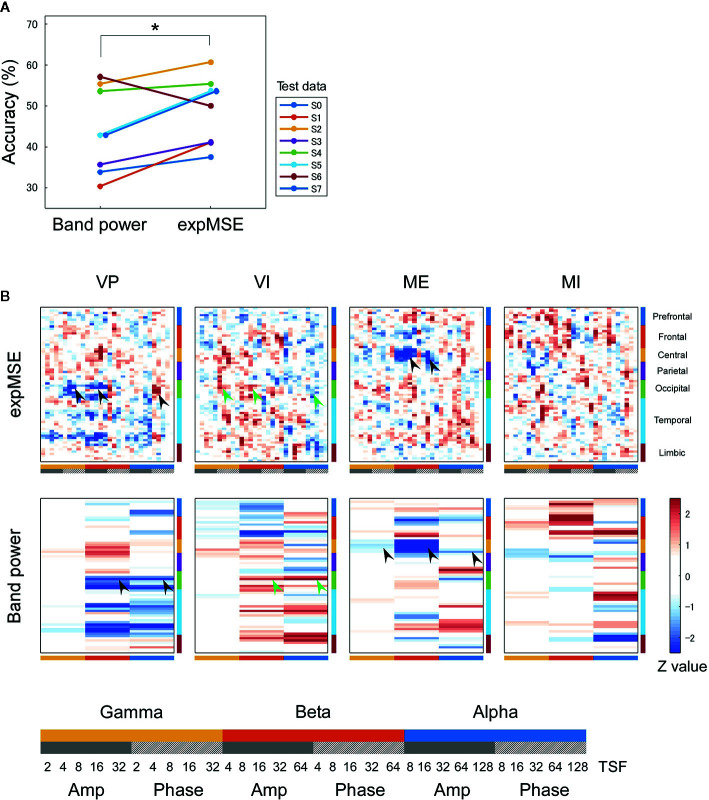
Inter-subject decoding of each task. **(A)** Decoding accuracies compared by band power and expMSE. Each color corresponds to a subject used for test data. *p < 0.05. **(B)** Task-related sensitivity maps. Upper and lower panels respectively portray maps for the expMSE and band power (VP, visual perception; VI, visual imagery; ME, motor execution; MI, motor imagery; black arrow, modality-specific sensitivity; green arrow, inverted sensitivity in the modality-specific regions). At the bottom are details of the horizontal axes: frequency × component (Amp/Phase) × TSF; Amp, amplitude expMSE; Phase, phase expMSE.

**Figure 5:**
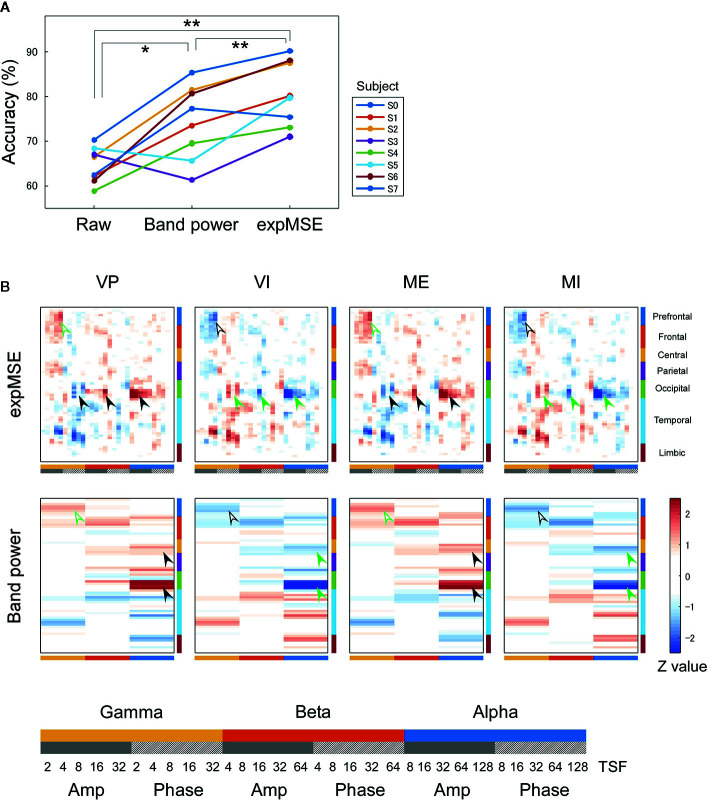
Intra-subject decoding of imagery behavior. **(A)** Decoding accuracies compared among raw data, band power and expMSE. Each color corresponds to a subject. *p < 0.05 and **p < 0.01. **(B)** Task-related sensitivity maps of example (subject S0). Upper and lower panels respectively show maps for the expMSE and band power (VP, visual perception; VI, visual imagery; ME, motor execution; MI, motor imagery; black filled arrow, modality-specific sensitivity; green filled arrow, inverted sensitivity in the modality-specific regions; black open arrow, modality-independent sensitivity; green open arrow, inverted sensitivity in the modality-independent regions). At the bottom are details of the horizontal axes: frequency × component (Amp/Phase) × TSF; Amp, amplitude expMSE; Phase, phase expMSE.

**Figure 6:**
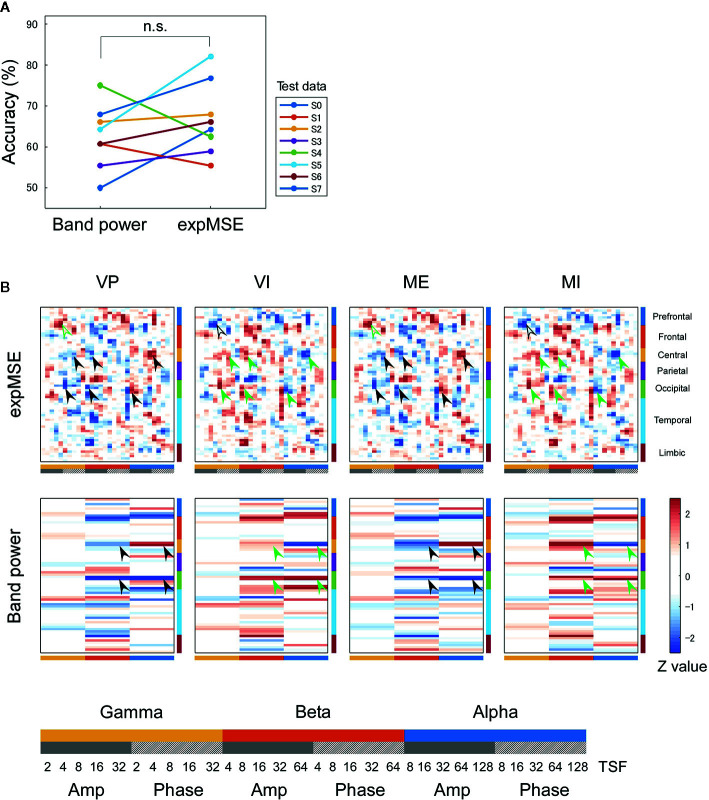
Inter-subject decoding of imagery behavior. **(A)** Decoding accuracies compared by band power and expMSE. Each color corresponds to a subject used for test data. n.s., not significant. **(B)** Task-related sensitivity maps. Upper and lower panels respectively portray maps for the expMSE and band power: VP, visual perception; VI, visual imagery; ME, motor execution; MI, motor imagery; black filled arrow, modality-specific sensitivity; green filled arrow, inverted sensitivity in the modality-specific regions; black open arrow, modality-independent sensitivity; green open arrow, inverted sensitivity in the modality-independent regions. At the bottom are details of the horizontal axes: frequency × component (Amp/Phase) × TSF; Amp, amplitude expMSE; Phase, phase expMSE.

Then, we define a sensitivity map *M_c_*(*x*) as

$$M_{c}\left(x\right)\;=\;\frac{\partial S_{c}\left(x\right)}{\partial x}$$

where ∂*S*_*c*_(*x*) represents the derivative of *S_c_*. Therefore, *M_c_*(*x*) represents the amount of change in the class score when input *x* is perturbed. We used gradients() function from keras.backend. This gradient of the class score with respect to input *x* elucidates which features are influential for the final classification (68–70). Intuitively, the larger the positive sensitivity feature is, and the smaller the negative sensitivity feature is, the more likely it is to be classified as the class. However, two points are noteworthy. The gradient is a derivative at each input. For that reason, the sensitivity might vary nonlinearly with the input value. Furthermore, the relation with other features is considered by the MVPA approach. Consequently, each sensitivity map represents the features of interest in each input, but unlike image recognition, there is less need to consider shift-invariance. Moreover, the differences between the inputs in each task are regarded as small. Therefore, the average maps of all inputs in each task are shown (**Figures 3B**, **4B**, **5B**, **6B** and **Supplementary Figures 3 **and **5**). In intra-subject decoding, we computed a sensitivity map for each input and standardized it by dividing it by the standard deviation within each map. The mean of all the maps in each task was used as the sensitivity map for each task for each subject (**Figures 3B** and **5B** and **Supplementary Figures 3** and **5**). In inter-subject decoding, we computed the map for each input and standardized it by dividing it by the standard deviation within each map. The mean of all inputs in each task was used as the sensitivity map for each task (**Figures 4B** and **6B**).

## Results

### Decoding of Multi-Modal Tasks

We first decoded the VP, VI, ME, and MI tasks, i.e., performed four-class classification. In intra-subject decoding, the expMSE showed higher performance than raw data and band power (raw, 48.3 ± 5.7%; band power, 68.2 ± 7.7%; expMSE, 79.0 ± 3.0%; *p*(band power – raw) < 0.001; *p*(expMSE – raw) < 0.001; *p*(expMSE – band power) = 0.003; **Figure 3A**). Similarly, in inter-subject decoding, the expMSE showed higher performance than band power (band power, 44.0 ± 10.4%; expMSE, 49.1 ± 8.3%; *p* = 0.048; **Figure 4A**). Additionally, in both intra-subject and inter-subject decoding with the expMSE, negative sensitivities in the gamma and beta band and positive sensitivities in the alpha band were observed in modality-specific regions (VP, occipital regions; ME, central regions) in non-imagery tasks (VP and ME, black filled arrows in **Figures 3B** and **4B** and **Supplementary Figure 3**). Inverted sensitivities were observed in imagery tasks (VI and MI, green filled arrows). Similarly, inverted sensitivities were found with band power.

### Decoding of Modality-Independent Imagery Behavior

Furthermore, we decoded modality-independent imagery behavior: we applied two-class classification (imagery vs. non-imagery). For intra-subject decoding, the expMSE showed higher performance than band power (raw, 64.6 ± 4.0%; band power, 74.3 ± 8.3%; expMSE, 80.6 ± 7.3%; *p*(band power – raw) = 0.019; *p*(expMSE – raw) < 0.001; *p*(expMSE – band power) = 0.006; **Figure 5A**). For inter-subject decoding, no significant difference of decoding performance was found between the expMSE and band power (band power, 62.5 ± 7.7%; expMSE, 66.7 ± 8.9%; *p* = 0.27; **Figure 6A**). In both intra-subject and inter-subject decoding with the expMSE, negative sensitivities in the higher frequency bands (gamma and beta) and positive sensitivities in the lower frequency bands (beta and alpha) were observed in modality-specific regions (VP, occipital regions; ME, central regions) in the non-imagery tasks (VP and ME, black filled arrows in **Figures 5B** and **6B** and **Supplementary Figure 5**). Inverted sensitivities were observed in the imagery tasks (VI and MI, green filled arrows). Additionally, negative sensitivities in the gamma amplitude were observed in the prefrontal regions in the imagery tasks (VI and MI, black open arrows in **Figures 5B** and **6B** and **Supplementary Figure 5**). Inverted sensitivities were observed in the non-imagery tasks (VP and ME, green open arrows). Similarly, inverted sensitivities were found with band power in the modality-specific regions. However, only one subject (S0) in the intra-subject decoding showed imagery-related sensitivities in the prefrontal regions with band power (**Supplementary Figure 5**).

## Discussion

Mental imagery involves modality-specific regions and modality-independent top-down inputs (38, 42, 47–50, 71). It has a multifaceted relation to psychiatric disorders related to patient abilities, symptoms and treatments (3, 36–38, 72). For this study, we used complexity analysis to examine neural oscillations that occur during mental imagery. It has been fruitfully applied for investigating neural oscillations involved in various psychiatric disorders (18, 20, 21, 23, 24, 26, 27, 73).

### Neural Decoding With expMSE

We have proposed expSampEn and expMSE to compute the complexity at each time point. The proposed measures are assumed to capture short-time complexity fluctuations. As shown in **Tables 1**–**4**, the conventional temporal complexity measures (ApEn and SampEn) and the new complexity measure (expMSE) were correlated significantly with the amplitude and on short time scales, suggesting that the standardized index across all 68 regions worked well, at least on the amplitude and short time scales.

Subsequently, neural decoding was performed using the complexity information of region × frequency × component (amplitude and phase) × time scale. For this study, this information was reshaped as a two-dimensional map of the 68 regions × 30 oscillatory properties. It was processed by shallow CNN (**Figure 2**), similarly to the method reported by Tayeb et al. (14). In addition, the raw data and band power were used as inputs for comparison. Similar models were used for them so that they could be compared under as similar conditions as possible (see *Inputs* and *Decoding*). For intra-subject decoding, the decoding performance was higher such that the expMSE > band power > raw data, with significant differences between conditions, in two classes of imagery and non-imagery and four-class (VP, VI, ME, and MI) classification (**Figures 3A** and **5A**). Although the raw data are regarded as having the greatest amount of information, the data were probably too complex to be learned in shallow CNN. Frequency decomposition is regarded as effective for extracting useful features for neural decoding (74–77). In addition, Tayeb et al. (14), used a model with two additional layers instead of frequency decomposition. Although band power extracted frequency information in advance to make it easier to learn than raw data, the expMSE has improved decoding performance, probably because of the additional time scale information. In other words, the complexity measure extracted useful features for neural decoding, suggesting the importance of the time scale information. However, the inter-subject decoding accuracy was lower than that of intra-subject decoding, probably because of individual differences (**Figures 4A** and **6A**). Earlier studies with different tasks also revealed a decrease in accuracy of approximately 10–20% (78, 79). Therefore, these results seem reasonable. The differences in the band power and expMSE were also smaller for inter-subject decoding than for intra-subject decoding. The difference for four-class classification was found to be significant by a *t*-test (*p* = 0.048) but not by a Wilcoxon signed rank test (*p* = 0.078). However, the accuracies exceeded chance levels in all cases except for one condition (S0 as test data in **Figure 6A**) in this study (chance levels were 25% for four-class classification and 50% for two-class classification). Reportedly, the accuracy was improved along with the increase in the number of participants providing training data (78). For that reason, future analyses must be conducted with greater numbers of participants.

### Imagery-Related Neural Oscillations

Mental imagery-related brain regions have been discussed in terms of modality-specific regions and supramodal imagery-related regions (39). To examine these regions, we drew sensitivity maps showing the gradient of each feature and the class score of each task. This method is used mainly in the field of image recognition to ascertain which pixels are used for classification. For this study, we applied this method to neural decoding and examined which regions and frequencies are the basis for classification.

We first consider intra-subject decoding with the expMSE. Practically, in the four-class classification shown in **Figure 3B** and **Supplementary Figure 3**, the characteristic sensitivities in the modality-specific regions (i.e. the occipital in VP and central in ME) were observed in VP and ME in many subjects (black filled arrows), with inverted sensitivities in VI and MI (green filled arrows). However, the supramodal imagery-related sensitivities were apparently observed only in S0 and S3 (black and green open arrows). This relation was similar for two-class classification (**Figure 5B** and **Supplementary Figure 5**). Although many subjects showed characteristic sensitivity in modality-specific regions (black and green filled arrows), only three subjects (S0, S1, and S3) appeared to show characteristic sensitivity in supramodal regions (black and green open arrows). Regarding both visual (38–40, 49, 50) and motor (39, 41, 42, 44, 45) results, reports of some fMRI studies have described that the primary sensorimotor cortices are less activated during imagery than during non-imagery, but the associative areas adjacent to the primary sensorimotor cortices are similarly activated during both imagery and non-imagery. Reports of some E/MEG studies have described that beta band event-related desynchronization in the motor cortex was exhibited in both ME and MI, but the response was significantly less intense during MI (43, 45, 80). A few EEG studies of visual imagery have revealed increased gamma power in the visual cortex (81) and have demonstrated usefulness of alpha power for the classification of VP and VI (82). Reportedly, the bottom-up processes from the primary visual cortex during VP switch to top-down controls from PFC during VI (38, 48). Also, the pattern of connectivity among motor-associative regions is reportedly converted between ME and MI (46) in fMRI studies. These differences in activity patterns in modality-specific regions might have helped in both four-class and two-class classification. For supramodal regions, some subjects showed characteristic sensitivities only in the PFC in this study (S0 and S3 in **Supplementary Figure 3** and S0, S1, and S3 in **Supplementary Figure 5**). Although the PFC and parietal regions have been reported as supramodal imagery-related regions in many fMRI studies (38, 39, 41–43, 46–51), few E/MEG reports have described the involvement of supramodal regions in motor imagery (43, 83). The supramodal regions might have more intra-subject variation or might be activated for shorter periods of time than the modality-specific regions. For inter-subject decoding, similar sensitivities in the modality-specific and supramodal regions were found, but they indicated more random patterns than intra-subject decoding did (**Figures 4B** and **6B**). This result suggests that useful features for classification have not been extracted properly and sufficiently, probably because of functional (e.g. frequency and time scale) and spatial differences among subjects. In addition, negative sensitivities at higher frequencies are commonly found for modality-specific regions during non-imagery tasks and for supramodal regions during imagery tasks throughout all conditions (two-class/four-class classification × intra-/inter-subject decoding). These observations suggest that low complexity at higher frequencies represents activation in the region.

We consider a sensitivity map with band power. In all conditions, sensitivity maps with band power were similar to that with expMSE, but few characteristic features were found in supramodal regions (only S0 in **Supplementary Figure 5**). This relative lack of features is probably attributable to the loss of information about time scale. Negative sensitivity in ME and positive sensitivity in MI were found in the beta band in the motor cortex, consistent with earlier studies in which beta desynchronization was more significant in ME than in MI (43, 45, 80). Although increased gamma power was reported not only in VP but also in VI (81), no characteristic sensitivity was found in the gamma band in the visual cortex (**Figures 3** and **4** and **Supplementary Figure 3**).

Consequently, the sensitivity maps with the expMSE yielded imagery-related activity patterns in both modality-specific and supramodal regions, consistent with results of earlier studies. The similarity of patterns found in visual and motor modalities suggests a common mechanism for creating mental imagery of several modalities. Various psychiatric disorders associated with mental imagery have also been suggested as related to PFC (84–87). As it has in the present study, investigating higher brain functions such as mental imagery might expand our physiological and pathological understanding of psychiatric disorders and facilitate the search for their biomarkers. Furthermore, neural decoding might help treat psychiatric disorders by application of its detailed results to neuromodulation methods such as neurofeedback (3–12) and transcranial stimulation (88, 89).

### Limitations

An important limitation for consideration in this study is its sample size. Typically, decoding research involves more than 100 trials per task (13, 14). For this study, the number of trials per task was reduced to 14 because the task design included various tasks for complexity analysis and the decoding of hierarchical behaviors. However, high decoding performance was obtained in the intra-subject decoding despite the small sample size (**Figures 3** and **5**). As described above, one possible reason for the high performance is that we used the temporal change of complexity as an input for decoding. Although hundreds of trials are regarded as reasonable for MI decoding from EEG and MEG (16), Foldes et al. (90) reported high decoding performance with less than 30 training trials for MEG decoding of ME and the resting state. This result might be mainly attributable to the design classifying substantially different modality (i.e. ME and the resting state) and partly because of the high spatial resolution of MEG compared to that of EEG (16, 90). The present study also performed MEG decoding of substantially different tasks (i.e. visual/motor and imagery/non-imagery). They are probably the reasons why we were able to decode with a small number of trials. Although this is only a pilot study with a small number of subjects, our technique seems to be valuable for extending our knowledge of the neural bases of hierarchical functions in the human brain. Therefore, further studies with larger sample size will be needed to warrant our findings.

An important strength of MEG is its high temporal resolution. Some earlier studies have benefited from the temporal resolution to examine the time evolution of neural processes of approximately tens to hundreds of milliseconds (91–93). Reports of some studies using fMRI have described increases in the effective connectivity from the supramodal regions to the visual associative regions during VI (48) and differences in the effective connectivity among motor-related regions during ME and during MI (46). These findings suggest that the supramodal regions are active before the modality-specific region are activated. Some models that account for the time evolution of neural processes might improve the decoding performance and advance the physiological interpretation.

In interpretation of the trained model, the sensitivity maps of inter-subject decoding were particularly unclear (**Figures 4** and **6**). Furthermore, although earlier studies have implicated medial and superior PFC as involved in modality-independent mental imagery (49, 71), the sensitivity map in this study was not able to detect precisely what area in the PFC was related to mental imagery. These shortcomings might be partly attributable to individual differences (see Section 4.2), but other influences must also be considered. Although we have visualized the trained models with the highest accuracy, accuracy and interpretability do not always correlate (94, 95). The present study has demonstrated the improvement of decoding accuracy achieved by using complexity measure as an input, but when considering its interpretation, it might be better to apply some constraint to the interpretability, rather than merely improving the accuracy.

Although MEG recordings were taken during multimodal visual, auditory and motor tasks, we specifically examined visual and motor data for decoding because acoustic stimuli were used as a cue of the start and the end of all tasks: we considered the effects of the cues. Future efforts must use a redesigned study to examine common neural oscillations among more variational modalities.

## Conclusion

This study compared the decoding performance of inputs of three types in neural decoding (raw data, band power, and expMSE) and used these methods to examine modality-specific and supramodal imagery-related neural oscillations. Results indicate the usefulness of CNN with the expMSE for neural decoding and support the possible common imagery-related mechanism proposed in earlier studies demonstrating that the modulated activity pattern in the modality-specific regions and the additional activity in the supramodal imagery-related regions might be involved in imagery behaviors.

## Data Availability Statement

The datasets presented in this article are not readily available because of a non-disclosure agreement. Requests to access the datasets should be directed to Naoki Furutani, furutaninaoki@gmail.com.

## Ethics Statement 

The studies involving human participants were reviewed and approved by the ethics committee of Kanazawa University Hospital. The patients/participants provided their written informed consent to participate in this study.

## Author Contributions

NF designed the study. NF, YY, HH, CH, and TI conducted data curation. NF and YN conducted data analyses. TT and MK supervised the research. NF and HI wrote the first draft of the manuscript. TT and MK revised the manuscript. All authors contributed to the article and approved the submitted version.

## Funding

This work was supported by the Center of Innovation Program and CREST (Grant Number JPMJCR17A4) from the Japan Science and Technology Agency (https://www.coistream.osaka-u.ac.jp/en). The funder had no role in the study design, data collection and analysis, decision to publish, or preparation of the manuscript.

## Conflict of Interest

The authors declare that the research was conducted in the absence of any commercial or financial relationships that could be construed as a potential conflict of interest.
