# Supplementary material for: Neural Decoding of Multi-Modal Imagery Behavior Focusing on Temporal Complexity
Source: Front Psychiatry. 2020 Jul 30;11:746. doi: 10.3389/fpsyt.2020.00746 (PMC7406828; doi:10.3389/fpsyt.2020.00746)

Supplementary Material

# Supplementary Figures and Tables

## Supplementary Tables

**Supplementary Table 1.** Decoding accuracies with filters of three types in the intra-subject four-class classification (%). Conv1 and Conv2 respectively denote convolutional filters of the first and second layers.

| Conv. filter size | Conv1 | 5 × 5 | 68 × 1 | 68 × 5 |
| --- | --- | --- | --- | --- |
|  | Conv2 | 5 × 5 | 1 × 5 | 1 × 5 |
| Subject | S0 | **79.0** | 71.7 | 70.5 |
|  | S1 | **78.7** | 76.7 | 74.8 |
|  | S2 | **81.6** | 77.5 | 77.3 |
|  | S3 | **78.3** | 77.4 | 75.5 |
|  | S4 | **74.5** | 70.2 | 63.2 |
|  | S5 | **81.6** | 79.2 | 71.9 |
|  | S6 | 83.0 | **83.2** | 80.4 |
|  | S7 | 75.4 | 75.4 | **75.5** |

## Supplementary Figures

**Supplementary Figure 3.** Sensitivity maps of intra-subject four-class decoding with expMSE and band power: VP, visual perception; VI, visual imagery; ME, motor execution; MI, motor imagery; black filled arrow, modality-specific sensitivity; green filled arrow, inverted sensitivity in the modality-specific regions; black open arrow, modality-independent sensitivity; green open arrow, inverted sensitivity in the modality-independent regions. At the bottom are details of the horizontal axes (frequency × component (Amp/Phase) × TSF: Amp, amplitude expMSE; Phase, phase expMSE).


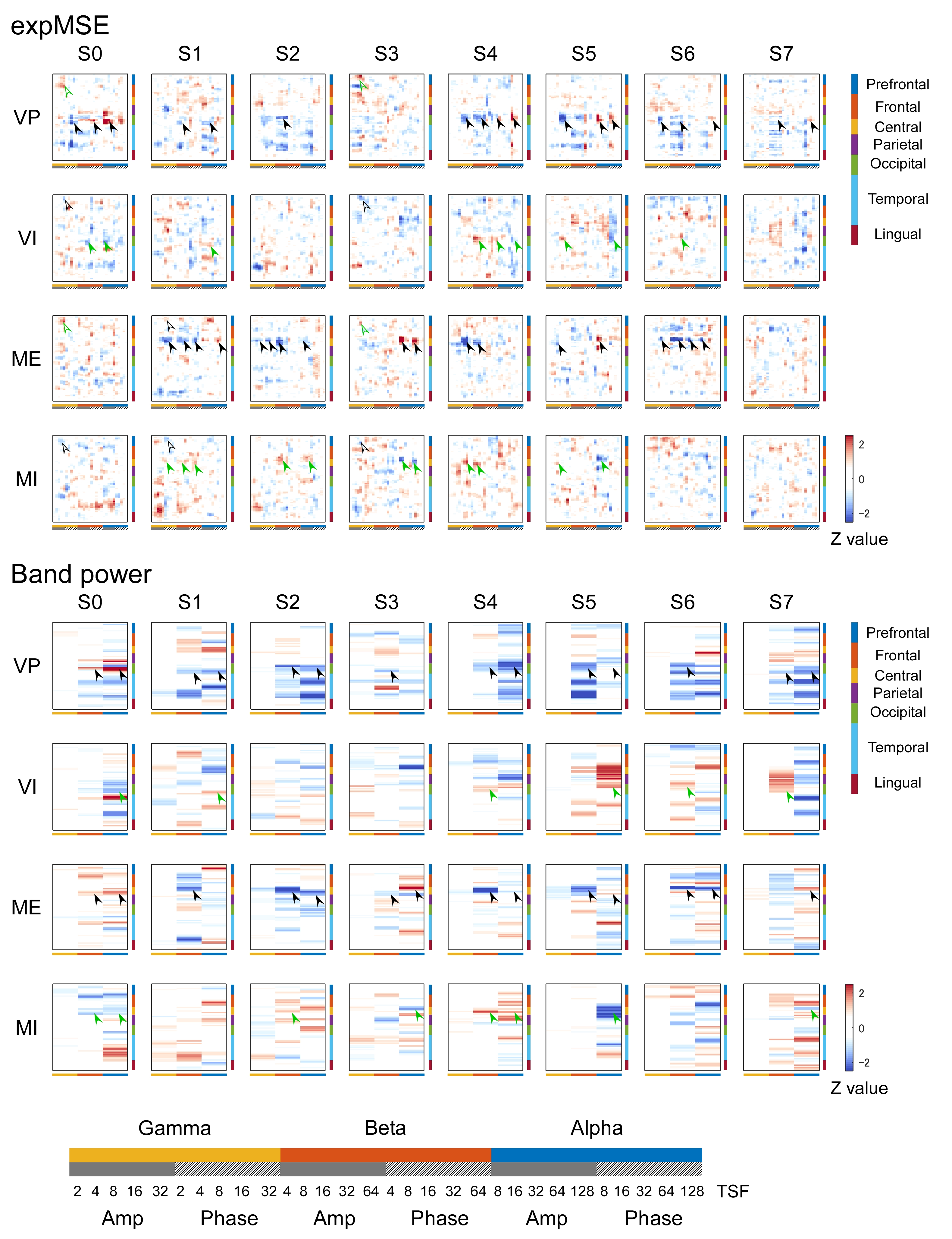


**Supplementary Figure 5.** Sensitivity maps of intra-subject imagery decoding with expMSE and band power: VP, visual perception; VI, visual imagery; ME, motor execution; MI, motor imagery; black filled arrow, modality-specific sensitivity; green filled arrow, inverted sensitivity in the modality-specific regions; black open arrow, modality-independent sensitivity; green open arrow, inverted sensitivity in the modality-independent regions. At the bottom are details of the horizontal axes (frequency × component (Amp/Phase) × TSF: Amp, amplitude expMSE; Phase, phase expMSE).


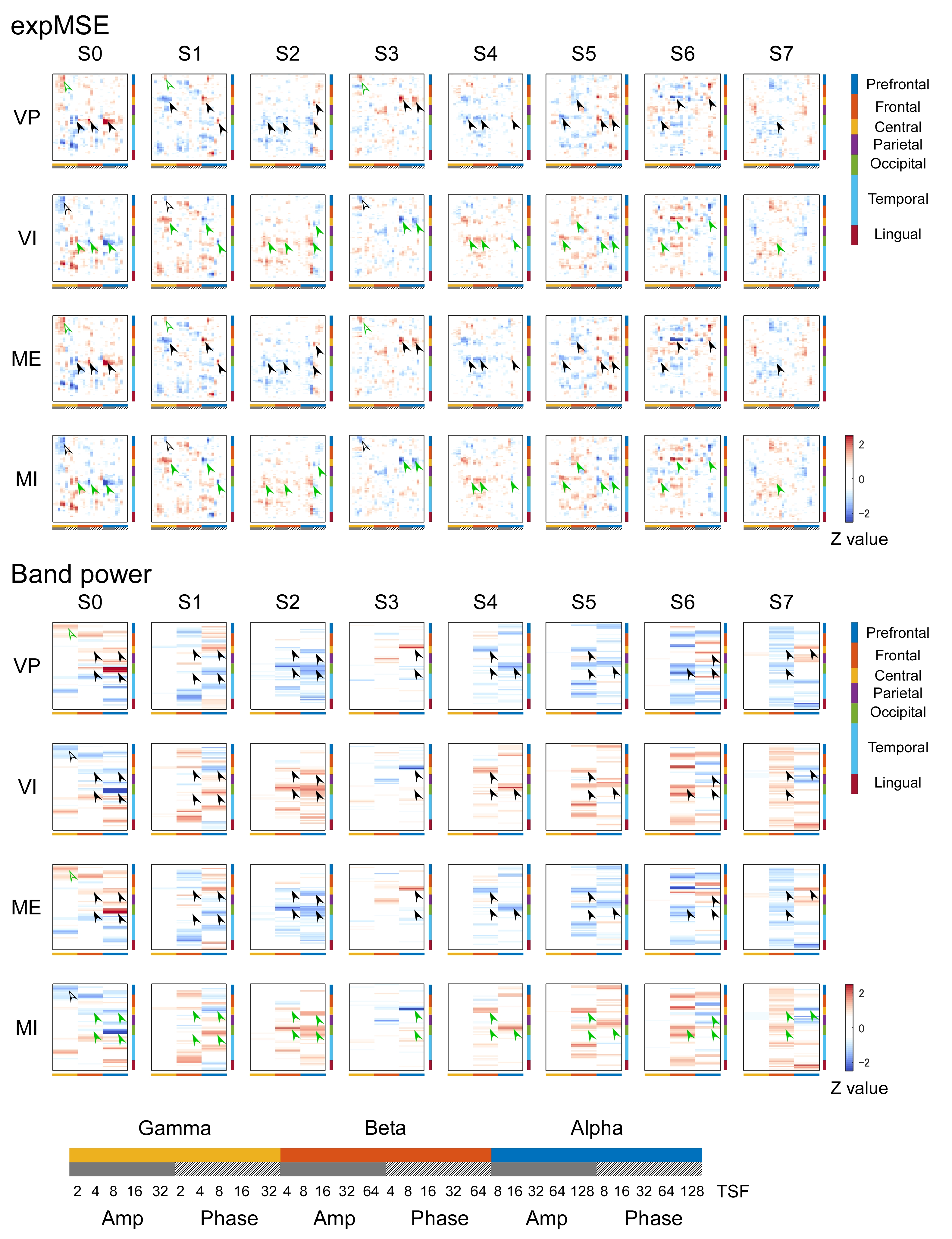

Supplement: Supplementary file 1 [file DataSheet_1.docx]
